# A Winter in the Upper Engadine, 1885
*The previous article appeared on December 20, 1913.


**Published:** 1914-01-24

**Authors:** 


### II. A WINTER IN THE UPPER ENGADINE, 1885.*

### By One of the First Consumptives to Visit the High Alps.

Many were the jokes made on the way home over the various experiences of the day. Quite an *esprit de corps* was maintained by the old hands as to "style" in skating. Our young friend the clergyman, who meekly ventured to assert that he could do the "outside edge," was brusquely met with: "What sir! do the *outside edge*! You must let the leg that you are not using hang gracefully down behind the other, not stick it out behind you like a broom-stick"; at which the mild youth for the moment collapsed.

When the surface of the Statzer-See was pretty well cut up, we were able to enjoy several days' delightful skating on the Sils Lake, one of the principal of the almost continuous chain of lakes which runs from St. Moritz to the Maloja Pass. There was an immense extent of ice here which was as perfect as possible. The only drawback to our enjoyment was that very often a keen wind swept along the valley, from which, in consequence of the exposed position of the lake, one was not able to get any shelter. It had one advantage, though, for it was often possible, by extending the flaps of one's great coat sail-wise, to be propelled from one end of the ice to the other, perhaps a distance of over a mile, without the slightest exertion. It is needless to say, though, that the return journey was not quite such an easy matter. These days were agreeably varied by the most pleasant picnic lunches, to which hot coffee, provided by an enterprisng Engadiner, was a most acceptable addition, and which were partaken of with appetites sharpened by the deliciously exhilarating atmosphere. There is, indeed, said to be a peculiar virtue in the air which has come into contact with this Alpine ice. Certain it is that most of the party seemed to feel little, if any, fatigue after their day's exertions. This lake-skating lasted for about a fortnight, when it was put an end to by a fall of snow which covered the ice to a considerable depth. Winter seeming about to set in in real earnest, the English visitors, who now mustered at the hotel to the number of about eighty, appointed two committees, one to superintend and provide outdoor amusements, the other to attend to the indoor amusements.

### The Strange Sport of Tobogganing.

In addition to skating, "tobogganing" is the great thing to be done out of doors. Of this I may as well at once give some explanation. A toboggan is nothing more nor less than a small sledge on iron runners, on which you descend snowy slopes. You sit upright with your legs stretched out in a horizontal position in front of you and your feet protruding beyond the curved ends of the runners. The seat is elevated about one foot above the ground, and is generally three feet in length and just broad enough to sit on comfortably. It has a cushion or not, according to fancy. Cords are attached to an iron bar connecting the points of the runners, by means of which the toboggan is hauled up to the starting point after making a descent. The toboggan "runs" are simply narrow courses of beaten-down snow, constructed with several twinings and twistings; for in the guiding of the machine the skill of tobogganing principally consists. For this purpose you are provided with two wooden sticks, each about two feet in length, tapering towards the point.

Loops of string are inserted in the handles through which you put your wrists, so that if, as not infrequently happens during a descent, you involuntarily slacken your hold, the sticks are not left far behind you peacefully reposing on the course, in which case there is often no alternative but to turn your machine askew into the deep snow with your feet, which almost invariably results in a "spill." The sticks are held pointing outwards and rather backwards, the slightest touch on the hardened snow of the course being sufficient to cause the toboggan to trend round either to the right or left, according to the stick brought into play. Speed in tobogganing largely depends on "pegging"—that is to say, striking the sticks, which are held firmly in a downward position, with momentary sharp jerks into the snow. "Pegging," of course, can only be indulged in when the toboggan does not for the time being require guiding. It very materially increases the momentum of the machine by the leverage exercised. I may add that "pegging" is the means of getting the toboggan into motion in the first instance.

There were two "runs" at St. Moritz. The racing run was nearly a mile in length. It started from a point rather behind and considerably above the hotel, and extended to the small village of Celerina on the road to Pontresina. It commenced with a very steep declivity, but after the first fifty yards became less steep, and continued pretty much on the same incline till the finish. The sides of it were well banked up with snow, to keep the toboggans from leaving the run. A separate foot-path was cleared in the snow near this course to enable the tobogganers who had reached the finish to walk up to the starting point again without blocking the run. This run was made for the special use and behoof of the English visitors, and the committee saw that it was kept in order. There was also the village run of about half a mile, which passed for some distance down the principal and only street of the place and then turned off down towards the St. Moritz Lake. This was open to everybody, and largely made use of by the villagers.

I found the exertion of dragging the machine up to the starting point after having made the journey down was very considerable, but this was more than compensated for by the delightfully exhilarating feeling of whizzing through the air, sometimes at the rate of over twenty miles an hour. The necessary spirit of emulation in the sport was kept up by means of races, which were frequently got up by the visitors amongst themselves and gave rise to much amusement. The ladies were quite as ardent tobogganers as the sterner sex, and generally entered for most of the races.

The starting point when a race is about to be held presents a curious spectacle to the uninitiated. The competitors are all assembled with their machines, which they have, so to speak, "in tow" by the cords I have described. As the contest is invariably decided by "time," all start separately. Their names are called out by an umpire one by one. Each racer as his turn approaches disposes himself comfortably in his toboggan, which is held in position at the scratch by another umpire. At the signal to start he commences "pegging" violently, and the machine soon begins to move swiftly away. On approaching the various turns in the course he shows his skill by carefully guiding the toboggan and, if necessary, slackening speed, so as to avoid the chance of its leaving the run; but on nearing the goal, where the course runs pretty straight, he increases his speed by a constant and vigorous use of the pegs, and as he

* The previous article appeared on December 20, 1913.

glides between the goal-posts the time is taken by a third umpire. Sometimes there were as many as fifteen or twenty entries for a race. A three minutes' interval was usually allowed between each start; but it sometimes happened that an exceedingly slow competitor was caught up by the next racer, and had, accordingly, to retire from the course, there not being sufficient room to allow of two people passing each other. Racers were frequently unseated at some of the ugly turns in the run, and at these points little knots of their admiring, but less venturesome, friends were wont to congregate. On one occasion the racing run was extended, so that the latter part of it should descend a more than usually steep hill. It was most amusing to watch the expression of the racers as with clenched teeth and anxious faces they approached the dreaded hill. It happened that at the bottom of it there was a very sharp "dip" over which it was necessary to pass to reach the goal. This had been filled up with snow on the previous day, and consequently the first two or three racers passed safely over it, though only with the greatest difficulty. The rest were not so fortunate, for seventeen of their number were pitched off their machines at the fatal spot into deep snow, which rose in clouds around them, and out of which they crawled, pitiable but ludicrous objects—none the worse however for their tumbles.

An immense amount of amusement was afforded by tobogganing. Sometimes a lady who was not quite venturesome enough to attempt the journey alone would accept a gentleman's invitation to travel down behind him. She accordingly knelt at the back of the machine and clutched violently hold of her protector's shoulders, and thus, in this safe but slightly ridiculous position, would reach the goal in safety. Not infrequently several toboggans were joined together and went in a procession down the run. But woe to the man who in a weak moment consented to bring up the rear; for unless he could stick on to his toboggan with the pertinacity of an "old man of the sea," he would be sure to be unseated by the violent swaying of his machine to and fro as the procession wound its way round the sharp turns of the course.

Tobogganing was the favourite sport of the Engadiners. On Sunday afternoons men, women, and children were to be seen tearing down the village run, and the air rang with the cries of "Achtung! Achtung!"—the usual call to those ahead to clear the course. It was not unusual to see a woman take two children down with her, one in her arms and one in her lap. Often a man would take his dog as a passenger, the latter seeming rather to enjoy the ride than not. The sturdy Engadiners did not trouble themselves with sticks, but guided themselves in the most rough-and-ready way with their heels—and seldom, if ever, was it that they came to grief.

It may be said at once that tobogganing is not a dangerous sport if not rashly pursued. Accidents do, of course, occasionally occur, but are generally the result of carelessness in guiding the machine. If a person is thrown off, it is almost invariably into the deep snow at the side of the run, which usually breaks the force of the fall completely.

I must not forget to mention that after the advent of the snow, the devotees of skating were still able to pursue their sport, for there were two excellently constructed open-air ice rinks near the hotel, which were kept thoroughly clear of snow and flooded on alternate nights, so as to preserve a smooth surface. The scene at mid-day on these rinks was indeed somewhat startling to one who had not visited the Engadine in winter and accordingly formed an exaggerated notion of the cold. The snow was banked round all the edges of the rinks, thus forming an almost complete shelter from the wind, if there was any. The sun was usually so warm that one could not bear an overcoat, and ladies were even glad to bring their sunshades into requisition. Those who were not strong enough to skate were able to sit out by the rinks watching the skaters without feeling the cold; and this, although we were in the depths of winter!

The committee for indoor amusements did their duty right loyally. They, in the most energetic manner, hunted up those who were good musicians or actors, and pressed them into their service. Once we actually had a fancy ball, and readings, concerts, or theatricals were held once a fortnight, in which, from the very smallness and isolation of the community, the keenest interest was manifested, and which naturally afforded ample matter for discussion, and kept the spirits of the party from flagging during the long winter evenings.

For the purpose of entertaining your friends the now time-honoured "afternoon tea" was the universal medium; but then, from the circumstances of the case, it was a very much more friendly and less formal affair than at home. Nobody thought any the worse of you for asking them to come and see you in your bedroom—for few people indulged in the luxury of a sitting-room. Pianos, moreover, were generally to be found as an article of bedroom furniture. These entertainments were the most agreeable and sociable in the world. But so much in vogue were they that often, if you wished to make sure of your friend, you had to ask him seven or eight days before.

Though most of the visitors were more or less invalids, their appearance after the first few weeks of their stay was certainly not calculated to arouse alarm. Indeed, a stranger coming among them would have stoutly maintained that they were base deceivers; for all, ladies included, were deeply bronzed by the sun and robust-looking.

The winter season lasts from the end of October or beginning of November till March, but some visitors came merely to spend their Christmas holidays. A pleasanter place to spend a winter holiday could hardly be chosen—at least, for those who do not mind the cold and are prepared to enter into the delights of skating and tobogganing. The days of December and January are generally cloudless, with the most delightful of warm suns; though, of course, it is pretty cold in the shade. Christmas is kept with all the ceremonies indulged in at home, but there is just a dash of newness about them which is not amiss once in a way.

It was with real regret that our party broke up at the end of the season. We all felt that we had spent a most delightful winter together, and that most of us had derived immense benefit from the climate. Many were the hopes expressed among us that we should "meet again next year." It has not, unfortunately, been my lot to revisit St. Moritz. I shall never cease, however, to remember my visit there as one of the pleasantest experiences of my life.

---

The directors of the London County and Westminster Bank, Ltd., after making provision for bad and doubtful debts, transferring £150,000 to premises account, £100,000 to provident fund, and placing £250,000 to an investment depreciation account, have declared a dividend of 10⅝ per cent. (less income tax) for the half-year ended December 31 last, making a total distribution for the year of 21¼ per cent.

